# Promotion of angiogenesis and suppression of inflammatory response in skin wound healing using exosome-loaded collagen sponge

**DOI:** 10.3389/fimmu.2024.1511526

**Published:** 2024-11-28

**Authors:** Siqi Zhang, Xugang Lu, Jun Chen, Shibing Xiong, Yifan Cui, Simeng Wang, Chongxia Yue, Qianqian Han, Bangcheng Yang

**Affiliations:** ^1^ Engineering Research Center in Biomaterials, Sichuan University, Chengdu, China; ^2^ NMPA Key Laboratory for Quality Research and Control of Tissue Regenerative Biomaterial & Institute of Regulatory Science for Medical Devices & NMPA Research Base of Regulatory Science for Medical Devices, Sichuan University, Chengdu, China; ^3^ Medical Device Testing Institute, National Institutes for Food and Drug Control, Beijing, China

**Keywords:** exosomes, collagen sponge, angiogenesis, inflammatory response modulation, wound healing

## Abstract

Effectively promoting skin wound healing remains a significant challenge in the medical field. Although stem cell-derived exosomes show potential in tissue regeneration, their local delivery and sustained release face challenges. To address these issues, we developed a collagen sponge based on type I and recombinant humanized type III collagen. Our study confirmed that exosomes were successfully loaded onto the sponge (sponge-Exo) and the sponge-Exo gradually released exosomes into the local milieu. The sponge-Exo played a crucial role in promoting the transition of macrophages from an inflammatory M1 phenotype to a regenerative M2 phenotype. Moreover, it enhanced the migration and proliferation of HDFs and promoted angiogenesis in HUVECs. Additionally, our findings revealed that the sponge-Exo accelerated wound healing by suppressing inflammatory response and stimulating angiogenesis in a rat full-thickness skin wounds model. Next generation sequencing (NGS) was used to explore the underlying mechanism of wound healing, and the results showed that the miRNAs (hsa-miR-21-5p and hsa-miR-29a-5p) associated with wound healing in exosomes were significantly up-regulated. These results highlight the remarkable effects of sponge-Exo on macrophage transformation, cell migration, proliferation and angiogenesis, which provide a potential prospect for the application in the field of skin wound healing.

## 1 Introduction

Skin wounds, particularly deep wounds in areas that move often, may have slow or insufficient healing, potentially causing infections that can weaken the skin’s important protective role and overall well-being (1, 2). Common medical methods for treating skin wounds mainly include surgical removal of dead tissue, negative pressure treatment, and regular dressing changes. However, these therapies frequently produce inadequate outcomes because of compromised cell functionality in the wound as it heals (3, 4).

The capacity of mesenchymal stem cells (MSCs) to differentiate into various cell types, combined with their remarkable anti-inflammatory attributes when present in wounds, has garnered increasing attention from researchers (5). Currently, many types of mesenchymal stem cells have been widely studied *in vitro* and *in vivo*, such as umbilical cord mesenchymal stem cells, bone marrow mesenchymal stem cells, and human dental pulp mesenchymal stem cells (hDPSCs) (6–9). Of these options, hDPSCs have numerous benefits, such as easy collection, strong ability to grow, and ability to differentiate into various cell types (10). Nevertheless, the transplantation of MSCs directly is constrained by various factors, including worries about atypical cell characteristics, inefficiencies in targeting specific areas, and alterations in the way cells develop and multiply (11). Scientists have developed different alternative methods to address the constraints of direct cell transplantation. In recent years, researchers have increasingly emphasized paracrine mechanisms as the primary therapeutic avenue for MSCs. They have illustrated that cell medium enriched with extracellular vesicles (EVs) can yield therapeutic outcomes similar to the direct application of MSCs (12–14).

Exosomes, tiny extracellular vesicles usually measuring between 30 and 150 nanometers, carry a variety of bioactive components, including miRNA, mRNA, lncRNA, circRNA, cholesterol, DNA and protein. Among them, miRNA can target and regulate the expression of specific genes in the recipient cells, thus playing a role in post-transcriptional regulation and affecting the biological processes of cells, such as migration, proliferation and differentiation (15, 16). Exosomes play a crucial role in intercellular communication by aiding in the transfer of membrane and cytosolic proteins, lipids, and RNAs between cells (17). Recent research suggests that exosomes from different types of stem cells can greatly enhance the healing of wounds and support the regeneration of skin through the stimulation of cell growth and movement, improved blood vessel formation, encouragement of skin cell layer regrowth, and regulation of immune reactions. The results highlight the possibility of exosomes as a viable substitute for stem cell therapy (18, 19). Despite their potential, intravenous injection of exosomes is hindered by rapid clearance through blood circulation, leading to off-target accumulation in the liver, spleen, and lungs, which significantly impact the efficacy and safety of exosome-based therapies (20, 21).

The challenge of rapid exosome clearance can be effectively addressed by developing scaffold materials, and there are many materials currently used to load exosomes, such as porous titanium scaffolds (22), hydroxyapatite (23) and PLGA (24). The exosomes loaded with these materials tend to release exosomes rapidly at the initial stage, which may affect the therapeutic effect. Compared with other materials, collagen scaffold possess intricate three-dimensional polymer structures that can prevent the initial burst release of exosomes, ideal for loading exosomes and supporting wound healing within moist environments (25). Type I and type III collagen are predominant components of skin, together make up approximately 80-85% and 15-20% of the total collagen composition in human skin (26). Both type I and type III collagen serve pivotal functions in maintaining the structural integrity of the skin and its underlying tissues (27). Current studies have shown that collagen scaffolds formed by type I and type III collagen have good biocompatibility (28).

This study focuses on developing an innovative collagen sponge loaded with exosomes to achieve sustained release of exosomes for enhanced wound healing. Exosomes are isolated from hDPSCs and incorporated into a collagen sponge that mimics the natural collagen components of the skin to improve local delivery efficiency. This system is designed to slow release of exosomes, thereby promoting cell proliferation, migration, and angiogenesis related to wound healing, ultimately enhancing the healing process. Additionally, next-generation sequencing is employed to analyze the expression of small RNAs in exosomes, aiming to uncover the mechanisms underlying their role in accelerating wound repair.

## 2 Materials and methods

### 2.1 Cell culture

This study was conducted in strict accordance with the document approved by the Ethics Committee of the West China Hospital of Stomatology, Sichuan University: WCHSIRBD-2019-040. The cells were cultured in a carbon dioxide (CO_2_) incubator at 37°C with 5% CO_2_ and a humidified atmosphere. Initially, hDPSCs were cultured in a proliferation medium composed of dulbecco’s modified eagle medium (DMEM, Gibco), 10% (v/v) fetal bovine serum (FBS, Gibco), 100 U/mL penicillin G, and 100 mg/mL streptomycin until they reached approximately 90% confluency.

### 2.2 Exosomes isolation, purification and identification

Exosomes were isolated from the conditioned medium of hDPSCs *in vitro*. Exosome purification involved multiple centrifugation and filtration steps. In short, the conditioned medium was spun at 500 g for 10 min and at 2000 g for another 10 min to get rid of cells, then spun at 10,000 g for half an hour, and finally passed through a 0.22 μm filter to remove any remaining cellular debris. Afterwards, the resulted supernatant was then processed using ultracentrifugation equipment from Beckman Coulter in the United States, spinning at a force of 100,000 g for 70 min, followed by a wash with phosphate-buffered saline (PBS) at the same force for an additional 70 min.

Transmission electron microscopy (TEM) was used to analyze the morphology of exosomes. Exosomes from hDPSCs were quickly treated with 4% paraformaldehyde for 10 min. Following this, around 8 mL of the combination was spread onto copper grids coated with carbon and left to dry in the air for 10 min. The grids were then stained twice with 4% phosphotungstic acid for 5 min. The TEM (HT7700, Hitachi) was used to perform imaging at 120 kV.

Moreover, exosomes were analyzed for particle size, concentration, and size distribution using Nanoparticle tracking analysis (NTA) with the Nanosight LM10 system in accordance with the manufacturer’s guidelines. NTA analytical software (Nanoparticle Tracking Analysis, version 2.3) was used to analyze the findings.

Exosomal markers were identified via western blotting (WB). Exosomes were first quantified for protein content using micro-BCA protein assay kit from Thermo Fisher Scientific in Rockford, IL. Then, equal amounts of proteins from cells and exosomes were lysed in ice-cold buffer containing a protease inhibitor cocktail. Finally, western blotting was performed to confirm the expression of CD63 (abcam) and β-actin (abcam).

### 2.3 Preparation of the collagen sponge and sponge-Exo

Type I Collagen was acquired by utilizing acetic acid extraction and pepsin digestion. Before collagen extraction, porcine skin samples were shaved of hair and outermost skin, finely cut, and treated with degreasers for 10 min at 40°C. The extracted crude collagen was then purified with 0.7 M NaCl solution after pepsin treatment for 2 hours. After purification, with 10000 g centrifugal 15 min, will be formed by particles dissolved in acetic acid concentration of 0.1 M and then at 4°C deionized water dialysis in 5 days.

To prepare collagen sponge, 8 mg of Col I suspension was mixed in 1mL of 2 mg/mL rhCol III (Jiangsu JLand Biotech Co., Ltd, China) solution. Next, a mixture of 20 μL of the 5% glutaraldehyde solution and 1 mL of collagen solution was cross-linked for 10 hours at room temperature. After crosslinking, the collagen hydrogel was cleaned with ultrapure water for 3 times, 5 min each time to remove free glutaraldehyde. The resulting hydrogel was then freezed at -20°C for 12 hours, followed by -70°C for 6 hours. Afterward, the collagen sponge was obtained by lyophilizing it for 48 hours with a vacuum freeze dryer from UNICRYO in Germany. Then 50 μg exosomes were added to the collagen sponge with 1.5 cm in diameter. Collagen sponge was kept at 4°C for 12 hours, and then cleaned with PBS to remove the free exosomes in the solution. Then, exosome-loaded collagen sponge (Sponge-Exo) was obtained.

### 2.4 Characterization of the collagen sponge and sponge-Exo

Gold was sprayed onto the collagen sponge and freeze-dried sponge-Exo, and their morphology and pore size were examined using a scanning electron microscope (SEM).

The swelling rate was evaluated by immersing collagen sponges and sponge-Exo in PBS at 37°C. At each time interval, the sponge was removed from the PBS to eliminate any excess liquid on the surface. The measurements were repeated until there was no significant increase of sponge weight. The swelling rate of the collagen sponge was calculated as:


$$Swelling\;rate\;\left(\%\right)=\frac{\left(M_{1}-M_{0}\right)}{M_{0}}\times 100\%$$

M_0_ was the collagen sponge’s initial mass, M_1_ was the weight of the collagen sponge after swelling.

The moisture retention rate was evaluated by placing fully moist collagen sponge and sponge-Exo in a 37°C incubator. The sponges’ mass was measured every 2 h and monitored for 14 h. The moisture retention rate of the sponges was calculated as:


$$moisture\;retention\;rate\;\left(\%\right)=\left[1-\frac{\left(m_{0}-m_{1}\right)}{m_{0}}\right]\times 100\%$$

m_0_ was the fully moist collagen sponge mass, m_1_ was the weight of the sponge after lost water.

Collagen sponge and sponge-Exo were immersed in PBS at 37°C to measure the degradation rate. They were weighed every 24h and monitored for 21 days. Remove moisture from the sponges’ surface before each weighing. The of the sponges was calculated as:


$$Degradation\;rate\;\left(\%\right)=\frac{\left(n_{0}-n_{1}\right)}{n_{0}}\times 100\%$$

n_0_ was the fully moist collagen sponge mass, n_1_ was the mass weighed after removing water from the sponge surface at each time point.

The presence of exosomes on the sponge was subsequently examined using confocal laser scanning microscopy (CLSM, Zeiss, LSM880, Germany) with exosomes labeled with the Dil cell membrane red fluorescent probe (Beyotime, China) (DiI is a lipophilic membrane dye that can diffuse laterally and gradually stain the cell membrane upon entry.). A sponge loaded with 50 µL of PBS was served as control.

The Horiba Fluorolog-3 Fourier transform infrared spectroscopy was utilized to examine the functional groups present in the collagen sponge.

The exosome release profile was assessed using a micro BCA protein assay kit. In brief, the prepared sponge-Exo, containing 50 μg of exosomes, was loaded into the upper transwell chamber within a 24-well plate. At the same time, 200 μL of PBS was introduced into the lower chamber. Following that, 20 μL of PBS was withdrawn and substituted with 20 μL of new PBS on days 0, 2, 4, 6, and 8. The exosome concentration was determined and the proportion of exosomes released was computed.

### 2.5 Cellular proliferation, migration, and angiogenesis assay

For cell proliferation, human dermal fibroblasts (HDFs) were seeded into 96-well plates at 3000 cells per well. The next day, collagen sponge extract, exosomes, and a combination of collagen sponge extract and exosomes were introduced into their designated wells. After 1, 3, 5, and 7 d of incubation, the supernatant was removed, fresh medium was added and 10% CCK-8 solution was added, and the optical density (OD) value was measured at 450 nm wavelength after 2 h of incubation. The experimental groups included the control group, collagen sponge group, exosomes group, and collagen sponge-Exo group.

Additionally, a scratch test was conducted to assess cell migration. Fibroblast cells were seeded in 24-well dishes at a concentration of 50,000 cells per well and left to grow for 24 hours. A sterile P-200 pipette tip was used to create a linear scratch on the cell monolayer. The debris was cleared, and the scratch edge was gently washed with PBS to smoothen it. Afterward, 200 μL of medium with 20 μg/mL exosomes replaced the original medium. Images were taken with an optical microscope and a digital camera (Olympus, Tokyo, Japan) was used for this task. The images were utilized to track the migration of cells towards the cell-free region at 5, 10 and 24 hours, respectively. Quantitative analysis of the reduced scratch area was conducted using the Image J analysis software (NIH, Bethesda, MD).

Cellular angiogenesis was assessed using the tube formation assay. Human umbilical vein endothelial cells (HUVECs) were seeded at a density of 1×10^4^ cells per well in a 48-well plate coated with Matrigel (BD Biosciences). The cells were then cultured with 20 μg/mL exosomes or an equivalent amount of PBS for 16 hours at 37°C. Following the incubation period, tube formation was examined using an optical microscope (Leica). ImageJ software was utilized to measure the overall size of the network structures.

### 2.6 Real-time quantitative polymerase chain reaction analysis

The expression of inflammation genes, including IL-10, TGFb, IL-1β, and TNF-α in RAW264.7 cells respectively treated with collagen sponge extract, exosomes, and a combination of collagen sponge extract and exosomes for 24 hours, was assessed using real-time qPCR. The reference gene employed was β-actin. TRIzol reagent (Accurate biology, Hunan, China) was utilized to isolate total RNA from RAW264.7 cells. Then, RNA samples with optical density ratios (260/280 nm) between 1.8 and 2.0 were reverse transcribed to generate cDNA. Real-time qPCR was performed using a 7500 Real-Time PCR Detection System (Bio-rad) and SYBR Green Master Mix (Accurate biology, Hunan, China). The primer sequences can be found in **Table 1**.

**Table 1:** List of primers used in this study for RT-qPCR.

*Gene*	*Forward (5′-3′)*	*Reverse (3′-5)*
*IL-10*	GAGAAGCATGGCCCAGAAATC	GAGAAATCGATGACAGGGCC
*TGFb*	CCAGATCCTGTCCAAACTAAGG	CTCTTTAGCATAGTAGTCCGCT
*IL-1β*	TGGAGAGTGTGGATCCCAAG	GGTGCTGATGTACCAGTTGG
*TNF-α*	CGCTGAGGTCAATCTGC	GGCTGGGTAGAGAATGGA
*β-actin*	CCTGAAGTACCCCATCGAGC	AGGGATAGCACAGCCTGGAT

### 2.7 Skin wound healing model in rats

The animal experiments were approved by the Ethics Review Committee of Sichuan University under the Ethics approval number KS2021535. A model of full-thickness excisional wounds was used to evaluate the wound-healing capability of collagen sponge and sponge-Exo. Randomly selected 12 healthy adult SD rats, according to the recovery time was divided into three groups (3 days, 7 days and 14 days), each group of four. To establish the wound model, the rats were anesthetized using 0.1 mL of 10% ketamine (vol/vol) and 0.05 mL of xylazine administered intraperitoneally. Their backs were shaved and disinfected with ethanol, then a 1.5 cm diameter circular wound was excised using curved scissors, resulting in three wounds on each rat’s back. The wounds were treated with collagen sponge or sponge-Exo. Only sterile gauze was used to treat the rats in the control group that did not receive the treatment. The dressings were then secured with a bandage. To study the reduction in wound size, macroscopic changes were observed and photographed at 3, 7 and 14 days post operation. The degree of wound reduction was quantitatively calculated using ImageJ software with the following equation:


$$wound\;closure\;\left(\%\right)=\frac{N_{i}}{N_{0}}\times 100\%$$

where N_0_ (mm) was the initial wound area, and N_i_ (mm) was the open wound area, respectively.

### 2.8 Histology analysis

At both the 7 and 14 days’ time points post-surgery, the rats were sacrificed and the skin of wound area was carefully removed, including the surrounding healthy skin, for histopathological analysis. To prepare the samples for analysis, the excised skin was fixed in 4% paraformaldehyde solution. Subsequently, a gradual dehydration process was conducted, followed by embedding the samples in paraffin and slicing them into sections that were approximately 4 µm in thickness. Hematoxylin and eosin (HE) staining was employed to assess the structure and characteristics of the tissues. Furthermore, collagen deposition and maturity in the wound bed were evaluated by Masson staining, and collagen deposition was quantified by ImageJ software.

### 2.9 Immunohistochemical staining for IL-6, CD31, and a-SMA

The tissue sections were dewaxed in water and immersed in a citric acid repair solution under high pressure for 5 min. To reduce endogenous peroxidase activity, the sections were treated with 3% hydrogen peroxide. Goat serum blocking solution was applied to the slides for 30 min to block non-specific binding. Then, the primary antibody was added to the sections dropwise and incubated overnight at 4°C. After washing with PBS, the sections were then incubated with the secondary antibody for 20 min in a dark room at room temperature. DAB chromophobe solution was applied for color development, and the reaction was stopped after washing with deionized water. Sections were counterstained with hematoxylin, dehydrated in ethanol, clarified in xylene, and sealed with neutral glue. Images of the slides were then captured using an Olympus BX53 microscope from Japan, and the analysis was conducted using ImageJ software. For the immunohistochemical (IHC) staining, three animals were examined per group. At least five fields were randomly selected for analysis in each sample. The data for IL-6, CD31, and a-SMA analyses were expressed as the percentage of positive cell numbers divided by the total cell numbers.

### 2.10 Exosome small RNA was detected by next generation sequencing

RNA from exosomes derived from hDPSCs was extracted using the RNA Isolation kit (Sigma-Aldrich). Then, next generation sequencing technology (Novogene, China) was used to detect the expression of. Low-quality bases were trimmed using Trimmomatic, and using FASTQC to verify the original fastq data. Chimirra was used for miRNA read counting, and miRNA expression was normalized by TMM values. Differentially expressed genes were identified by the edgeR program, and genes with a greater than 1.5-fold change in expression were considered differentially expressed genes.

### 2.11 Statistical analysis

All quantitative data were presented as means ± standard deviation (SD)(n≥3). Statistical analysis was performed using SPSS software, and significant differences were assessed using one-way analysis of variance (ANOVA). Statistical difference at *P< 0.05 or **P< 0.01 were considered statistically significant.

## 3 Results

### 3.1 Characterization of exosomes derived from hDPSCs.

**Figure 1:**
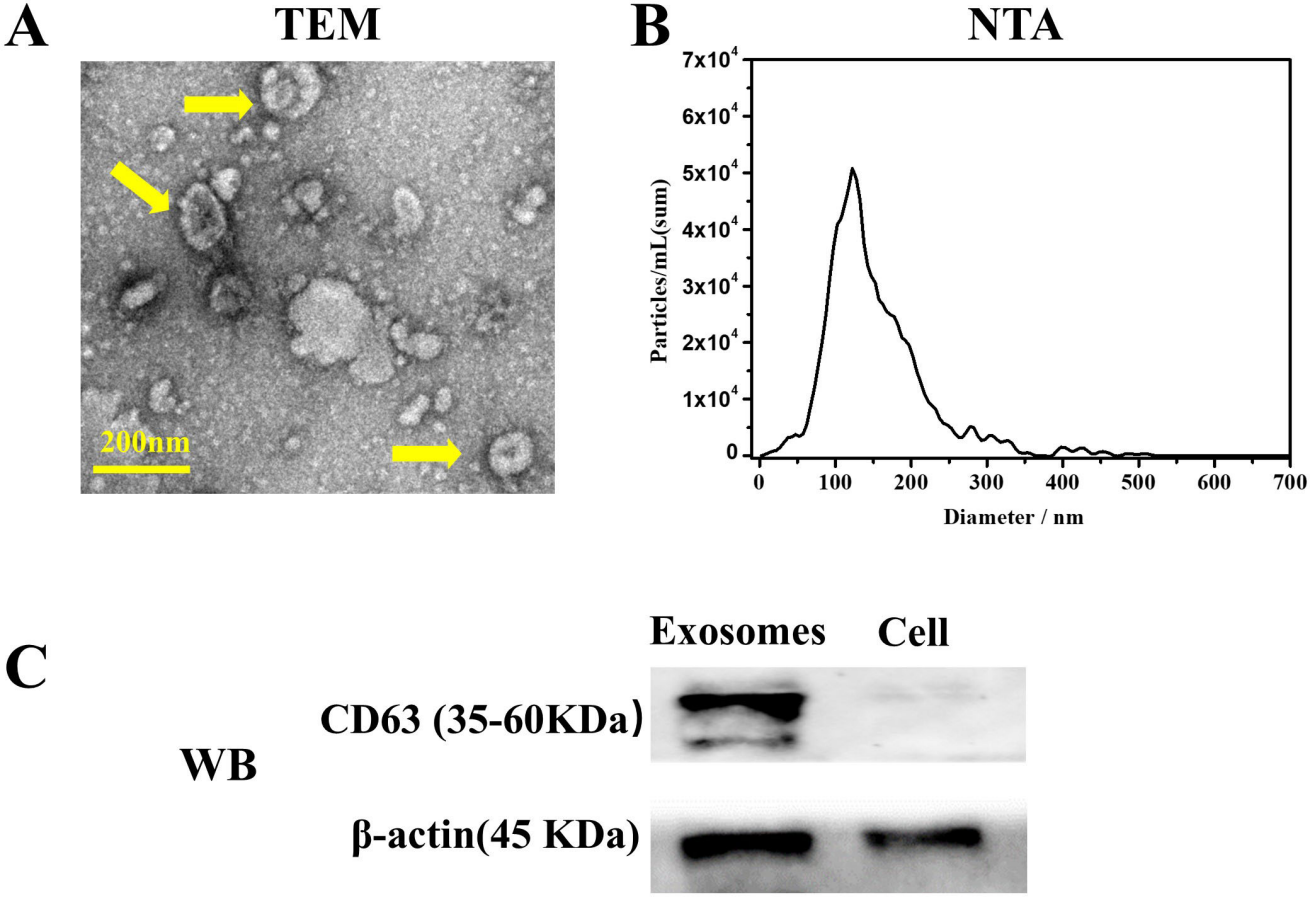
Characterization of exosomes derived from human dental pulp mesenchymal stem cells (hDPSCs). **(A)** Transmission electron microscopy revealed the distinctive cup-shaped morphology of exosomes (yellow arrow). **(B)** Particle size distribution analysis using Nanoparticle tracking analysis indicated the size of obtained exosomes ranged from 40 to 180 nm. **(C)** Western blot analysis confirmed the presence of exosomal surface marker CD63.

Transmission electron microscopy (TEM) analysis revealed the distinctive cup-shaped morphology of hDPSCs-derived exosomes (**Figure 1A**). Nanoparticle tracking analysis (NTA) indicated that the size of the exosomes ranged primarily from 40 to 180 nm (**Figure 1B**). Western blotting analysis (**Figure 1C**) confirmed the presence of exosome-specific marker CD63 in hDPSC-derived exosomes.

### 3.2 Characterization of collagen sponge

**Figure 2:**
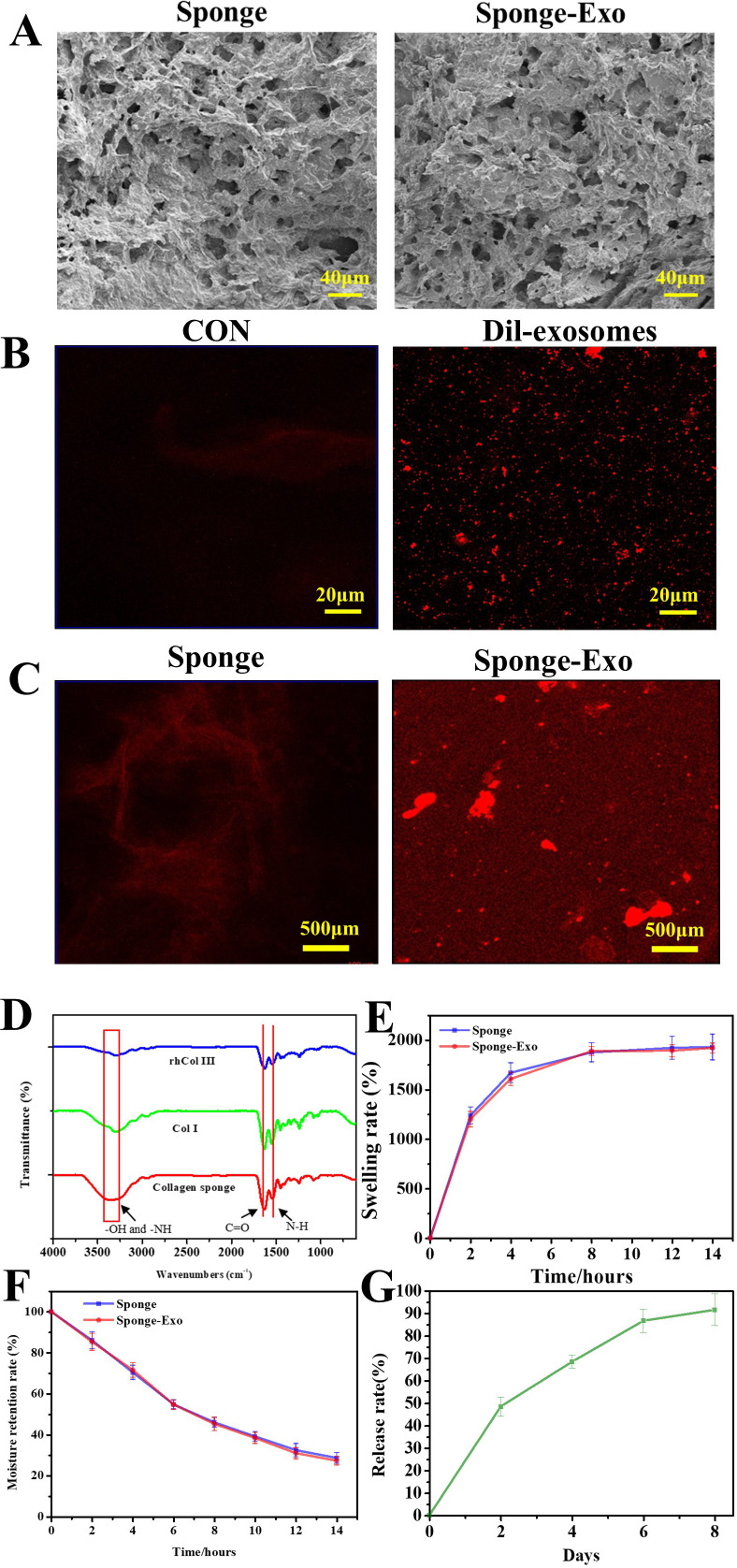
**(A)** SEM images of collagen sponge and sponge-Exo. **(B)** CLSM images of Dil-exosomes, CON represented the exosomes without Dil dye was added. **(C)** Detection of exosomes on the collagen sponge, CLSM images of the collagen sponge and collagen sponge-Exo. **(D)** FTIR spectra were used to analyze the groups of the collagen sponge. **(E)** The swelling rate and **(F)** moisture retention capacity of the collagen sponge and sponge-Exo were measured in PBS at different time points. **(G)** Sustained release of exosomes in the sponge-Exo.

SEM images in **Figure 2A** revealed the porous structure in the surface of the collagen sponge with pores size ranging from 20 to 50 µm. After the loading of exosomes, the pore size of the sponge did not change.

**Figure 2B** shows that exosomes could be successfully stained red by Dil dye. Compared to the collagen sponge (**Figure 2C**), the sponge loaded with Dil-labeled exosomes exhibited a greater amount of red fluorescence, confirming the presence of exosome particles.

The FTIR spectrum of the collagen sponge (**Figure 2D**) exhibited characteristic bands at different wave numbers. These bands corresponded to various molecular vibrations, such as N-H and O-H groups at approximately 3,288 cm^−1^, C = O stretching at 1623 cm^−1^, amide vibration overlapping with N-H bending vibration at 1523 cm^−1^, and C-H deformation vibration at 1440 cm^−1^. Collagen sponge had stronger amide vibration at 1523 cm^−1^ than collagen type III and collagen type I. These results indicate that the N-H and O-H groups of type III collagen and type I collagen react to form amide groups after the addition of glutaraldehyde, thus allowing the collagen to form a dense network structure.

**Figure 2E** illustrated notable swelling properties of the collagen sponge and sponge-Exo in PBS over time. After 12 hours, the sponge reached swelling equilibrium in PBS, with a solution absorbing capacity nearly 20 times its weight. In addition, the degradation of the sponge was tested and the sponge could not degradation over 21 days *in vitro* (results not shown).

Moreover, the sponge exhibited excellent moisture retention capacity (**Figure 2F**). It retained water for more than 12 hours and contained about 32.5% of the initial water volume after 12 hours.

Exosome release from the sponge-Exo occurred gradually, with about 86% released at 6 days (**Figure 2G**). The exosome membrane surface contains g-acyl group and ϵ-amino groups (29), which enable exosomes to form hydrogen bonds and electrostatic interactions with the -COOH and -NH2 groups present in collagen. This interaction effectively prevents the abrupt release of exosomes in a safe manner. These findings suggested that collagen sponge is capable of controlling the sustained release of exosomes, thereby ensuring the continuous action of exosomes within the sponge-Exo dressing at the wound site. This sustained release maintains a relatively stable concentration of exosomes, which potentially facilitates the repair of skin at the wound site.

### 3.3 Evaluation of migration, proliferation, and angiogenesis using collagen sponge and sponge-Exo *in vitro*

**Figure 3:**
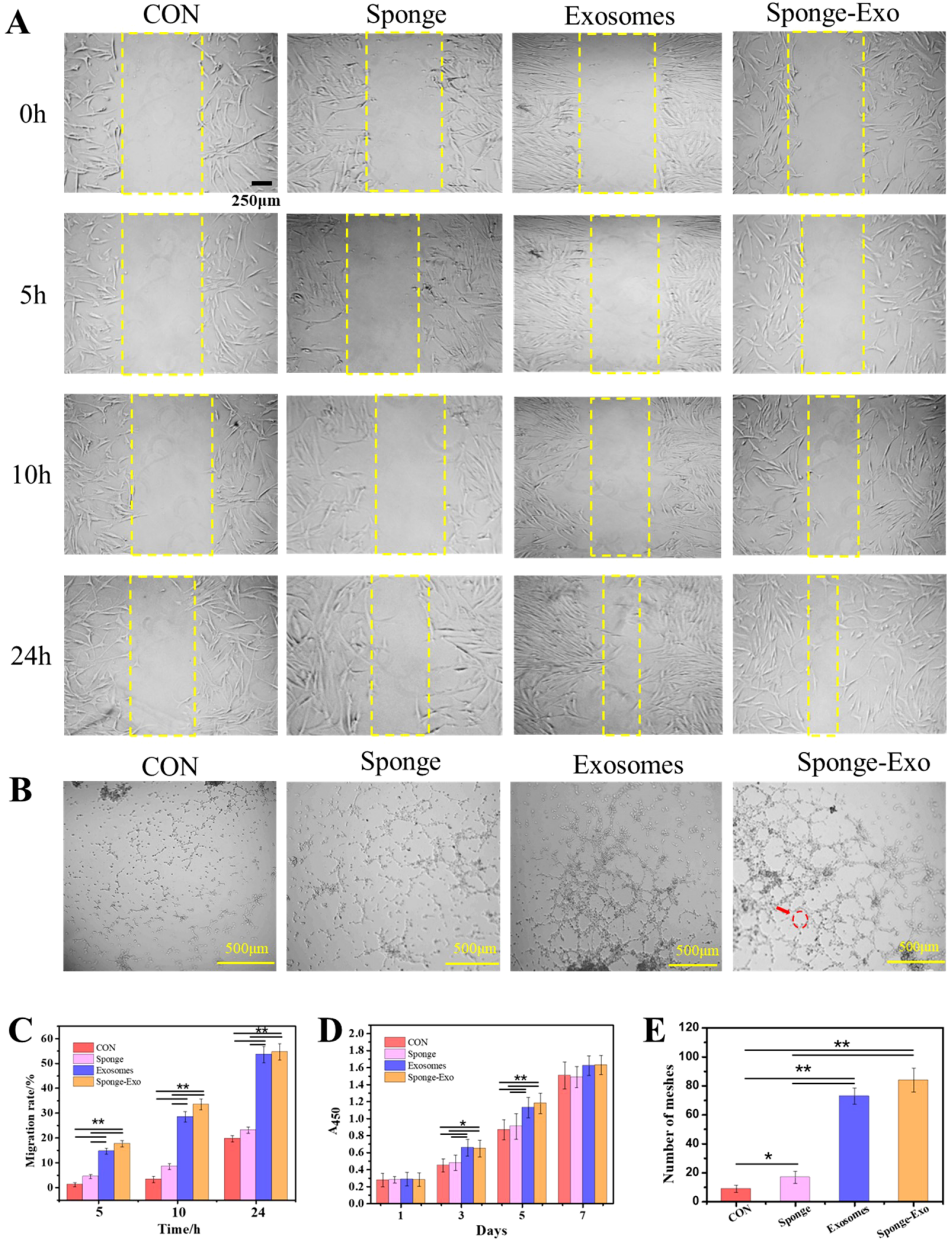
Sponge-Exo stimulated cell migration, proliferation, and angiogenesis *in vitro*. **(A)** Evaluation of the impact of sponge-Exo on HDFs migration through cell scratch assays. **(B)** HUVEC angiogenesis was studied by growing cells in medium containing sponge, exosomes, and sponge-Exo, respectively. The red arrow represents the mesh. **(C)** Quantitative analysis of migration rate of HDFs, **(D)** and HDFs proliferation through CCK-8 assays. **(E)** Quantitative analysis of the total meshes. The “*” represents p< 0.05, “**” represents p< 0.01.

To assess the migration, proliferation and angiogenesis effect of collagen sponge and sponge-Exo on different cells *in vitro*, we conducted CCK-8, scratch wound assay, and tube formation assays. Our findings indicated that exosomes greatly increased the migration of HDFs, as illustrated in **Figures 3A, C**. Moreover, the treatment with both exosomes and sponge-Exo at a concentration of 20 μg/mL markedly boosted the proliferation capabilities of HDFs at 3 days and 5 days (as depicted in **Figure 3D**). Moreover, the *in vitro* experiments showed that compared with the control group, the sponge group, the exosome group and the sponge-Exo group could all promote angiogenesis and increase the number of mesh (**Figure 3B, E**), among which exosomes and sponge-Exo had more significant ability to promote angiogenesis *in vitro*.

### 3.4 Cellular inflammation regulation by sponge-Exo

**Figure 4:**
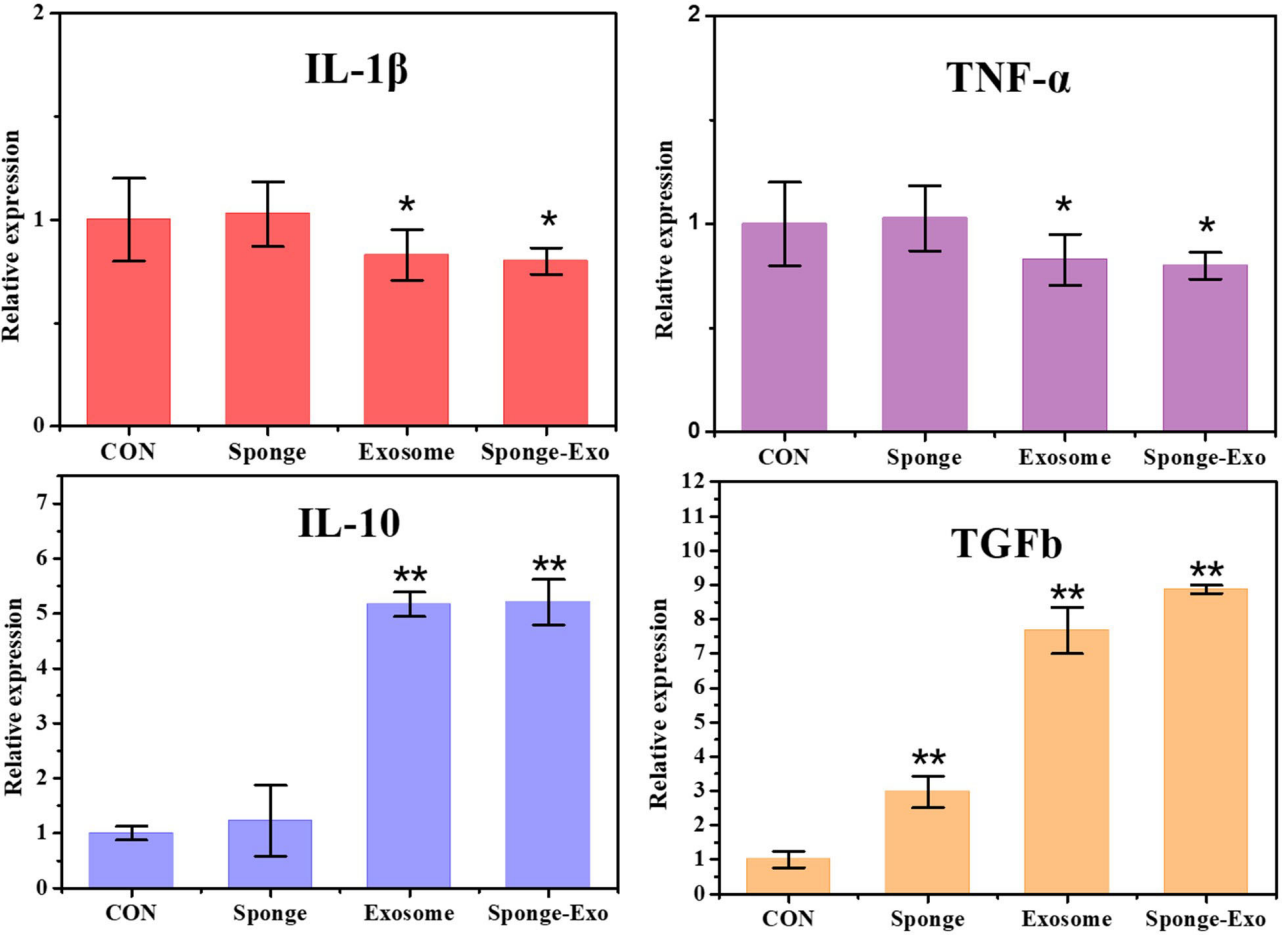
RT-qPCR was conducted to identify the presence of M1 and M2 markers in RAW264.7 cells. CON represents RAW264.7 cells that were not treated with exosomes or sponge. The “*” represents vs CON, p< 0.05, “**” represents vs CON, p< 0.01.

To evaluate the polarization impact of RAW264.7 cells, particular genes such as IL-1β and TNF-α were analyzed as indicators of M1-type macrophages, while IL-10 and TGFb were examined as indicators of M2-type macrophages. The findings indicated that sponge-Exo led to a significant decrease in the mRNA expression of pro-inflammatory genes IL-1β and TNF-α, along with a significant increase in the mRNA levels of anti-inflammatory genes IL-10 and TGFb (**Figure 4**). These findings indicated that the composite sponge-Exo effectively promoted the transformation of RAW264.7 cells into the M2 phenotype.

### 3.5 Evaluation of *in vivo* wound closure using collagen sponge and sponge-Exo

**Figure 5:**
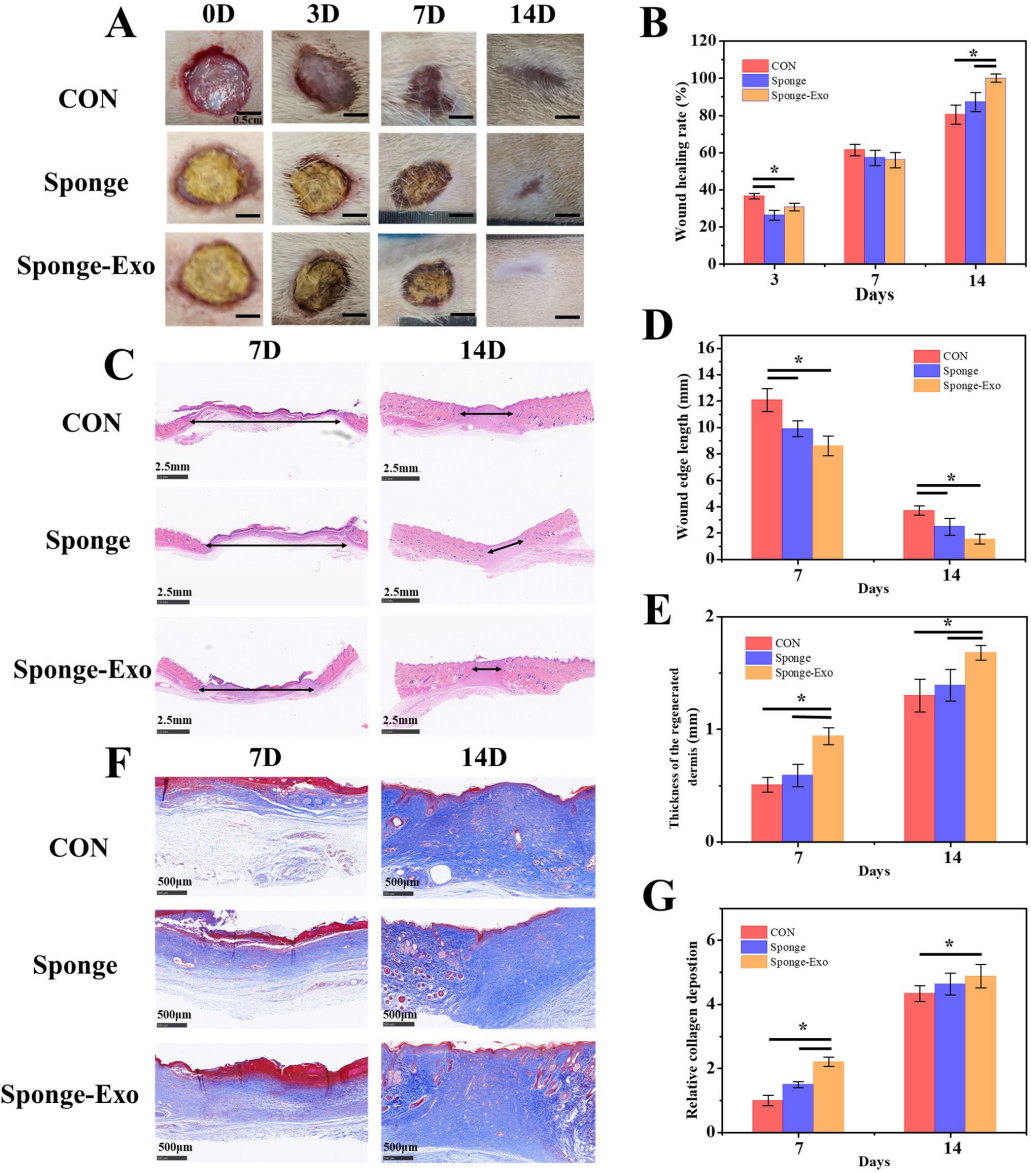
The effect of the collagen sponge and sponge-Exo on wound healing *in vivo*. **(A)** Images of wounds in different group at 0, 3, 7, and 14 days. **(B)** Quantitative analysis of wound healing rates at 0, 3, 7, and 14 days in different group. **(C)** HE staining of wound tissue in different group at 7 and 14 days, double-headed arrows indicated wound edges. Quantitative analysis of scar length **(D)** and thickness of regenerated dermis **(E)** in wound tissue. **(F)** Masson staining of wound tissue in different group at 7 and 14 days. **(G)** Quantitative analysis of collagen deposition in wound tissue. The “*” represents p< 0.05.

We assessed wound healing in three groups of rats: the control group, the collagen sponge group, and the sponge-Exo group. **Figures 5A, B** show that the rate of wound healing in both the sponge group and the sponge-Exo group was slower than that of the control group on the 3rd and 7th days. This difference was mainly attributed to the material completely covering the epidermis, preventing the closure of the wound epidermis. However, as the recovery time went on, after the material fell off with the blood scab, the wound healing rate of the sponge-Exo group and the sponge group was significantly better than that of the control group on the 14th day. By this time, the sponge-Exo group had successfully achieved a 100% rate of complete wound healing, while the healing rate of the control group was only about 80%.

Assessment of wound healing and regeneration progress was enhanced through measurements of decreased scar length and improved collagen maturity. At 7 and 14 days after the operation, the sponge-Exo group exhibited notable enhancements in the thickness of the regenerated dermis and narrower wound edges in comparison to the control and sponge groups (**Figures 5C-E**). Furthermore, the sponge-Exo group showed increased and more structured collagen deposition in the wounds when compared to the other groups (**Figures 5F, G**). These findings suggested that the sponge-Exo treatment significantly accelerated wound dermis regeneration and collagen deposition, leading to enhanced wound healing.

### 3.6 Cellular inflammation and angiogenesis of skin wound tissue

**Figure 6:**
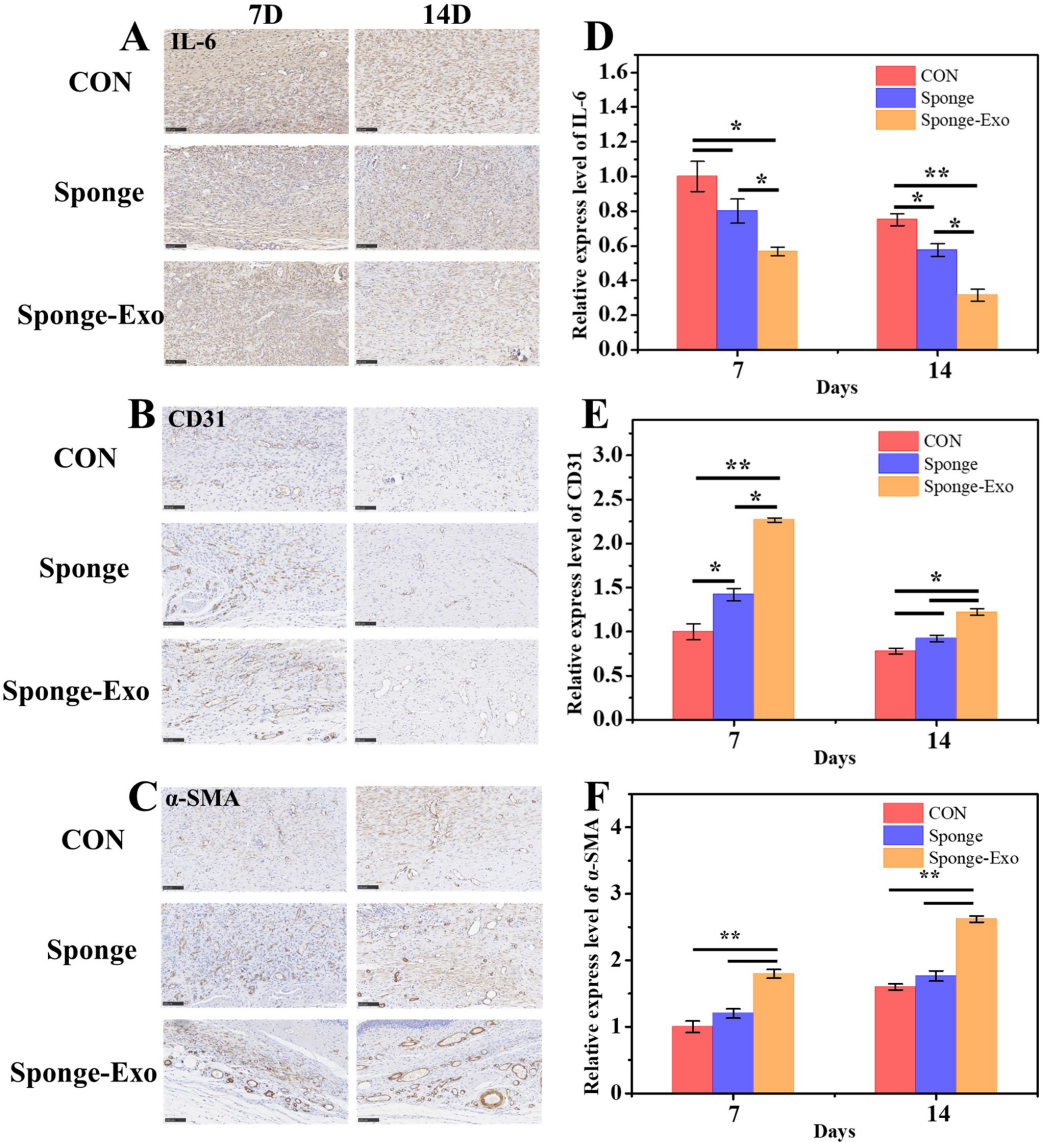
Histomorphological assessment of wound regeneration on cell inflammation and angiogenesis. Immunohistology staining was conducted on wound tissues treated with various collagen sponges for 7 and 14 days, targeting IL-6 **(A)**, CD31 **(B)**, and α-SMA **(C)**. Scale bar: 100 μm. Quantitative evaluation of IL-6 **(D)**, CD31 **(E)**, and α-SMA **(F)** levels after using the sponge and sponge-Exo for 7 and 14 days. The “*” represents p< 0.05, “**” represents p< 0.01.

The level of IL-6 expression is a crucial factor in assessing the inflammatory reaction of wound tissue (30). Immunohistochemistry results showed a notable decrease in IL-6 expression in the sponge-Exo group compared to the other groups on day 7 and 14, as depicted in **Figures 6A, D**. This discovery indicated that sponge-Exo was able to suppress the inflammatory reaction in the skin wound area.

Angiogenesis is crucial during the proliferation stage of wound healing, providing necessary nutrients and oxygen for cell growth (31). Immunohistochemical staining for CD31 and α-SMA was used to evaluate the development of new and mature blood vessels in the wound healing process. The sponge-Exo therapy for 7 days resulted in notably increased numbers of new microvessels and fully developed vessels in the regenerated flexible skin tissue, surpassing the control group. It is important to note that the sponge-Exo group showed an impressive characteristic-the formation of fully developed blood vessels (α-SMA) originating from the newly formed microvessels (CD31) by the 7th day. These mature vessels were densely deposited around fibroblasts or interwoven with functional glands, facilitating the provision of nutrition and oxygen (as seen in **Figures 6C, F**). In contrast, the collagen sponge groups solely displayed higher densities of neogenetic microvessels (CD31) in comparison to the control group (as depicted in **Figures 6B, E**). The findings suggested that exosomes released by the sponge-Exo were instrumental in speeding up the development of new microvessels in the elastic wound skin tissue. These microvessels rapidly matured into fully functional blood vessels within 7 days, a process significantly quicker than the sponge group, and the control group, which required at least 14 days.

### 3.7 Small RNAs analysis in exosomes

**Figure 7:**
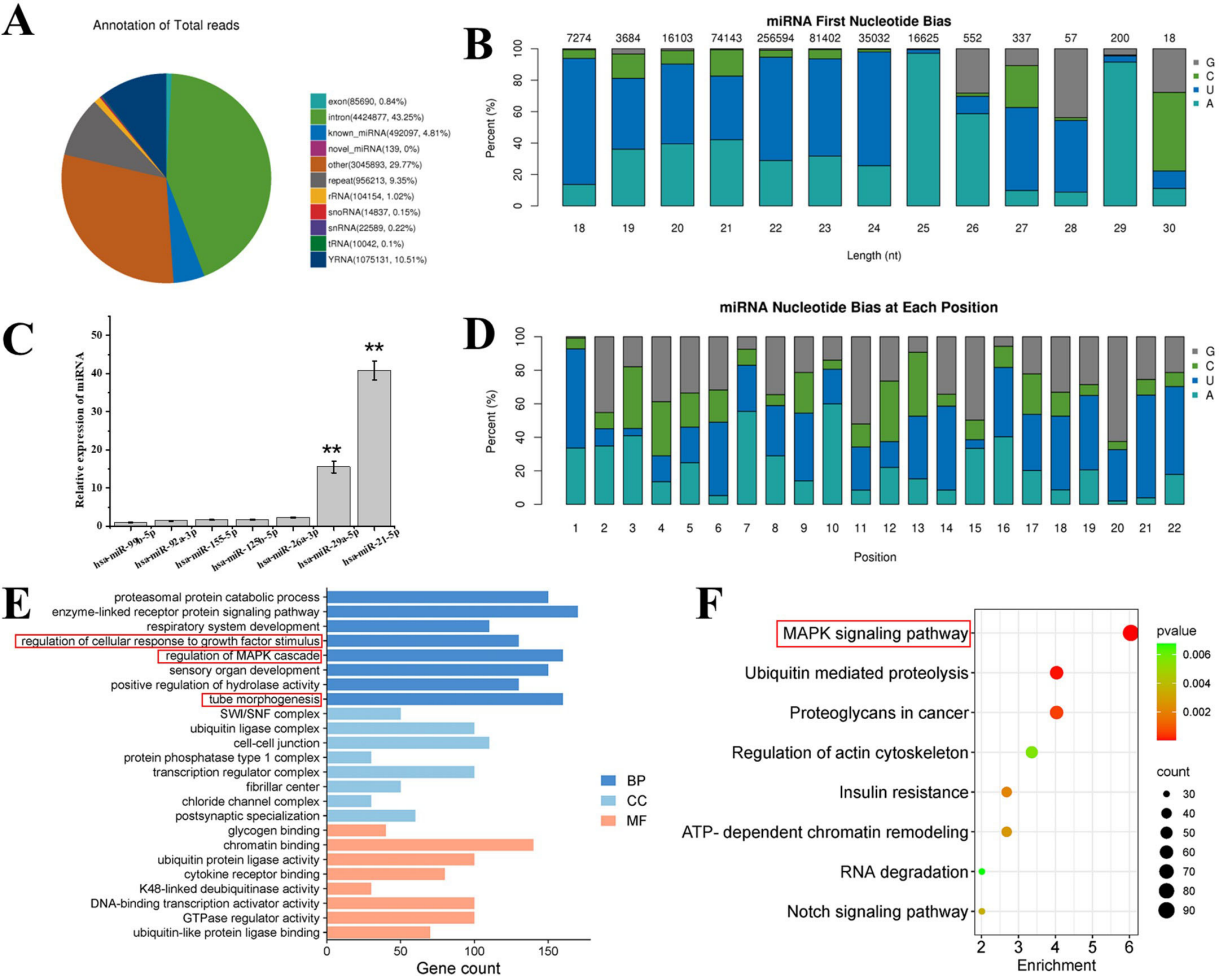
**(A)** Total category reads in exosome. known_miRNA: The number and proportion of sRNAs aligned to known miRNAs in each sample. RRNA tRNA/snRNA/snoRNA/YRNA: refers to the sample, respectively, compared to the rRNA/tRNA/snRNA/snoRNA/YRNA sRNA quantity and proportion. repeat refers to the number and proportion of sRNA aligned to repeat in each sample. **(B)** First base preference of known miRNA with miRNA lengths of 18—30 nt and base preference of each known miRNA in different exosomes. **(C)** Relative expression levels of wound healing related miRNAs in exosomes derived from hDPSCs through the next generation sequencing. **(D)** Different exosome base bias at each site of known miRNA. **(E)** GO analysis and **(F)** KEGG pathway analysis of hDPSCs derived exosome. BP, biological process; CC, cellular component; MF, molecular function. “**” represents p < 0.01.

The alignment and annotation of all small RNAs were be summarized. small RNAs were classified in the following priority order: known miRNA > rRNA > tRNA > snRNA > snoRNA > YRNA > repeat > gene > novel miRNA. As can be seen from d **Figure 7A**, hDPSC-derived exosomes contain a large number of introns, which play an important role in the transcription process, covering multiple stages such as transcription initiation, transcription elongation, transcription termination, polyA, and exonuclear transport, as well as the influence on mRNA stability.

In recent years, miRNAs have been extensively studied in the field of small RNA due to their unique targeting properties and other properties. Therefore, the miRNA base bias was analyzed. It could be observed from **Figure 7B** that for the miRNAs in exosomes, the first base preferences for miRNAs with lengths of 18 nt, 19 nt, 20 nt, 21 nt, 22 nt, 23 nt, 24 nt, and 28 nt were U; for miRNAs with a length of 27 nt, the first base preferences were U and G; for miRNAs with lengths of 25 nt, 26 nt, and 29 nt, the first base preference is A; and for miRNAs with a length of 30 nt, the first base is either C or G. Further, as depicted in the base preferences at each site in **Figure 7D**, for all the miRNAs in the alignment, bases at positions 1, 6, 9, 13, 14, 16, 17, 18, 19, 21, and 22 were mainly U, while the probabilities of C and G at the remaining sites were higher than that of A. These results indicate that the quality of the miRNA sequences in the alignment was relatively good.

The function of sponge-Exo in promoting wound repair mainly relies on the regulation of related cells by exosomes derived from hDPSCs after their release to the wound area. The expression of miRNAs related to promoting wound healing in exosomes was analyzed. As depicted in **Figure 7C**, the eight miRNAs with the highest expression levels related to wound healing were selected. It was discovered that both hsa-miR-21-5p and hsa-miR-29a-5p, which facilitate wound healing, were significantly higher than other miRNAs. Some miRNAs that inhibit wound healing (hsa-miR-99b-5p, hsa-miR-92a-3p, and hsa-miR-26a-3p) had relatively lower expression levels. It has been reported in the literature that miR-21 plays a crucial role in inflammation resolution (32). Additionally, miR-21 also exerts an important function during the proliferation phase. miR-21 has been proven to promote the migration of keratinocytes and fibroblasts. When miR-21 is inhibited, the re-epithelialization process is delayed, and the contraction of the damaged wound is also suppressed (33, 34). Moreover, miR-29a can reverse the pro-fibrotic phenotype of fibroblasts by down-regulating the expression of collagen and TIMP-1 (35). These results suggest that exosomes in sponge-Exo can deliver relevant miRNAs to the cells at the wound site to regulate their biological functions and promote wound healing and regeneration.

Go analysis (**Figure 7E**) of highly expressed miRNAs in hDPSCs derived exosomes showed that in terms of biological processes, genes in exosomes were mainly involved in regulation of cellular response to growth factor stimulus, regulation of MAPK cascade and tube morphogenesis. KEGG pathway analysis (**Figure 7F**) showed that exosomes might activate the MAPK signaling pathway, thereby promoting skin wound healing.

## 4 Discussion

With the advancement of regenerative medicine, the technology for repairing tissues and organs has significantly progressed compared to traditional methods. Seed cells, which are the cornerstone of tissue repair, are extensively utilized across various fields of regenerative medicine (36). However, their application is still challenged by issues such as low cell survival post-transplantation, reduced regenerative capacity, immune rejection, and ethical concerns (37). Consequently, the general and safe application of stem cell banks in regenerative medicine remains difficult. The paracrine effect of cells, particularly the secretion of exosomes, plays a crucial role in cell communication, immune response, angiogenesis, scar formation, tissue repair, and other biological functions (38–40). Exosomes are commonly present in cells, serum, and various other biological fluids. Consisting of tiny biological components like mRNA, miRNA, DNA, lipids, proteins, and metabolites, these substances are crucial for intercellular communication and can control numerous biological functions. They have garnered interest in various areas, including treatment for diseases, identifying biomarkers, and delivering medications (41). We performed Go analysis of highly expressed miRNAs on hDPSCs-derived exosomes and found that in terms of biological processes, genes in exosomes are mainly involved in regulating the response of cells to growth factor stimulation, regulating MAPK cascades and tube morphogenesis. KEGG pathway analysis showed that exosomes might activate the MAPK signaling pathway, thereby promoting skin wound healing. These results are similar to previous reports that the activation of the MAPK pathway by mesenchymal stem cell-derived exosomes (MSC-exosomes) in the process of skin repair (42).

MSC-exosomes were capable of delivering their contents to target cells, exerting significant regulatory functions (43). Exosomes have been shown to have positive effects on cells during skin wound healing, particularly in promoting cell proliferation and angiogenesis (44). However, direct injection of exosomes into the wound often leads to their inactivation and rapid metabolism by the body. To address this, we developed a sponge with type I collagen as a scaffold supplemented with recombinant humanized type III collagen to achieve sustained local release of exosomes. According to previous studies, the pore size of the sponge should be between 20-150 μm, which is beneficial for the loading of exosomes and is beneficial for cell proliferation and migration (45). The 3D structure of collagen dressings has ideal pores that facilitate water absorption and retention, providing a moist environment for the skin wound, ensuring the entry of nutrients and oxygen as well as the removal of metabolic waste products (46). The collagen sponge synthesized in this study has a pore size of 20 to 50 μm, which has a good exosome loading capacity. Our release profile data verified that the sponge-Exo successfully accomplished this sustained local delivery of exosomes. Notably, in the context of skin wound repair, by combining the functionalities of collagen and exosomes, the sponge-Exo offers distinct therapeutic advantages in comparison to the use of collagen sponge or exosomes alone. This innovative approach holds significant promise for wound healing applications of sponge-Exo.

The wound healing process involves distinct phases, including hemostatic, inflammatory, proliferative and remodeling stages, each needing precise orchestration in terms of sequence and timing (5). During the inflammation phase, a key element was the shift in macrophage polarization, especially as acute wounds progress to the regenerative M2 phenotype, known for its anti-inflammatory properties (47). The ability of the sponge-Exo to modulate the inflammatory response was of paramount importance for its clinical utility. Significant elevation was noted in the mRNA levels of IL-10 and TGFb, important indicators of M2-type macrophages, in the sponge-Exo group. Conversely, there was a notable decrease in the mRNA levels of IL-1β and TNF-α, which are important indicators of M1 macrophages. These results suggest that macrophage conversion to an anti-inflammatory phenotype can be effectively modulated by collagen sponge-Exo. Studies have shown that MSC-exosomes can modulate the equilibrium between M1 and M2 macrophages, thus halting the development and advancement of experimental inflammation in animal studies (48) Immunohistochemical results *in vivo* showed that sponge-Exo treatment significantly reduced the level of inflammatory marker IL-6 at day 7 and day 14, indicating that sponge-Exo treatment effectively prevented the development of inflammation. MSC-derived exosomes, which contain unique adhesion molecules like CD29, CD44, and CD73, have the ability to target damaged and inflamed tissues. Furthermore, MSC-derived exosomes showed a tendency to gather in the inflamed kidney and damaged brain in models of acute kidney injury and intracerebral hemorrhage, respectively (49). Additionally, research has shown that MSC-exosomes stimulate the transformation of M2 macrophages via the pKNOX1 pathway, leading to improved anti-inflammatory capabilities and accelerated wound healing (50). Aligning with these studies, our findings indicated that the potential to regulate inflammation of collagen sponge-Exo makes it clinically valuable for wound healing.

In the proliferative stage, the quick growth and movement of skin fibroblasts help create extracellular matrix, which aids in the healing of wounds (51). The findings from our study showed that the combination of sponge and exosomes promoted the growth and movement of HDFs more effectively than either sponge or exosomes individually. HE staining showed narrower wound area and thicker dermis in the sponge-Exo group than in the other groups at 7 and 14 days, indicating that sponge-Exo could still effectively promote the growth and migration of cells related to the wound site *in vivo*. Moreover, our findings also confirmed that the sponge-Exo significantly enhanced wound closure, promoted granulation tissue formation, and facilitated collagen deposition compared to the use of either sponge alone or the control *in vivo*. Besides managing inflammation and cellular migration, the effective transport of nutrients to the wound site significantly impacts wound repair. In our study, the incorporation of exosomes within the collagen sponge notably promoted the angiogenesis of HUVECs *in vitro*. Additionally, the sponge-Exo exhibited significantly increasing the expression of cell vascularization markers CD31 and α-SMA *in vivo*. The findings indicated that sponge-Exo had the potential to stimulate the formation of new blood vessels and improves the transportation of oxygen and nutrients to the injured area. Research indicates that exosomes containing high levels of VEGF-A can boost blood vessel formation in the damaged spinal cord and stimulate the regrowth of small blood vessels in mice suffering from spinal cord injuries (52). The present study demonstrated the significant impact of sponge-Exo on the biological characteristics of skin wound cells by examining its effects on inflammation, migration, and angiogenesis.

The complexity of cellular signaling pathways involved in wound healing suggests that exosomes may be involved in a wide range of biological processes, potentially affecting cell proliferation, migration, angiogenesis, and immune regulation. However, the current study may not be able to comprehensively cover all these complex interactions. Understanding the precise molecular mechanisms of exosomal RNA effects and identifying specific signaling pathways could significantly improve our understanding of their role in wound repair. Future studies should investigate the relevant signaling pathways in more depth, improve the design and use of exosomes-based therapies, and ensure more targeted and effective interventions for wound healing.

## 5 Conclusion

In summary, to optimize the application of exosomes in wound repair, we constructed a collagen sponge wound dressing that mimic the composition of skin collagen. The collagen sponge wound dressing based on type I collagen with the addition of recombinant humanized type III collagen, and loaded with hDPSC-derived exosomes. The sponge-Exo exhibited the capacity for sustained local release of exosomes, thereby significantly enhancing anti-inflammatory, proliferative, migratory, and tube formation capabilities of cells involved in the wound healing process. Furthermore, our novel collagen sponge dressing had proven effective in promoting wound closure and tissue regeneration in rats. Next generation sequencing (NGS) results showed that the miRNAs (hsa-miR-21-5p and hsa-miR-29a-5p) associated with wound healing in exosomes were significantly up-regulated, and KEGG pathway analysis showed that exosomes might activate the MAPK signaling pathway. In conclusion, the multifunctional sponge-Exo offered a sustainable means of exosomes release and accelerated the wound healing process, representing a promising and innovative approach for addressing wounds.

## Data Availability

The original contributions presented in the study are included in the article. Further inquiries can be directed to the corresponding author.
